# Approach to the Diagnosis and Management of Subarachnoid Hemorrhage

**DOI:** 10.5811/westjem.2019.1.37352

**Published:** 2019-02-28

**Authors:** Evie Marcolini, Jason Hine

**Affiliations:** * University of Vermont Medical Center, Department of Emergency Medicine, Burlington, Vermont; † Tufts University School of Medicine, Department of Emergency Medicine, Boston, Massachusetts

## Abstract

Headache is one of the most common reasons for presentation to the emergency department (ED), seen in up to 2% of patients.^1^ Most are benign, but it is imperative to understand and discern the life-threatening causes of headache when they present. Headache caused by a subarachnoid hemorrhage (SAH) from a ruptured aneurysm is one of the most deadly, with a median case-fatality of 27–44%.^2^ Fortunately, it is also rare, comprising only 1% of all headaches presenting to the ED.^3^ On initial presentation, the one-year mortality of untreated SAH is up to 65%.^4^ With appropriate diagnosis and treatment, mortality can be reduced to 18%.^5^ The implications are profound: Our careful assessment leading to the detection of a SAH as the cause of headache can significantly decrease our patients’ mortality. If this were an easy task, the 12% reported rate of missed diagnosis would not exist.^6^ We have multiple tools and strategies to evaluate the patient with severe headache and must understand the strengths and limitations of each tool. Herein we will describe the available strategies, as well as the ED management of the patient with SAH.

## INTRODUCTION

A 50-year-old female was preparing her children for school when she experienced a headache severe enough to make her lie down on the sofa. She managed to get the children off to school, but the headache did not abate. She was used to headaches, as she had migraines periodically that were controlled with over-the-counter medications, but this one was different and much more intense. She took a couple of acetaminophen, and when the pain was not relieved she brought herself to the emergency department (ED).

Headache is one of the most common reasons for presentation to the ED, seen in up to 2% of patients.^1^ Most are benign, but it is imperative to understand and discern the life-threatening causes of headache when they present. Headache caused by a subarachnoid hematoma (SAH) from a ruptured aneurysm is one of the most deadly, with a median case-fatality of 27–44%.^2^ Fortunately, it is also rare, comprising only 1% of all headaches presenting to the ED.^3^ On initial presentation, the one-year mortality of *untreated* SAH is up to 65%.^4^ With appropriate diagnosis and treatment, mortality can be reduced to 18%.^5^

The implications are profound: Our careful assessment leading to the detection of a SAH as the cause of headache can significantly decrease our patients’ mortality. If this were an easy task, the 12% misdiagnosis rate would not exist.^6^ We have multiple tools and strategies to evaluate the patient with severe headache, and must understand the strengths and limitations of each tool.

### Pathophysiology

Eighty-five percent of cases of atraumatic SAH result from a ruptured aneurysm.^7^ Alternate etiologies include perimesencephalic hemorrhage, which has a benign course, as well as arteriovenous malformations, dural arteriovenous fistula, arterial dissection, mycotic aneurysm, and cocaine abuse. The prevalence of aneurysms in the general population is roughly 2–5%,^8^ greater in those with family history of aneurysms, and/or personal history of Ehlers-Danlos or polycystic kidney disease. Not all aneurysms are dangerous. Factors associated with the risk of rupture include hypertension, tobacco use, excessive alcohol use, sympathomimetic drugs, Black race, Hispanic ethnicity, and aneurysmal size > 10 millimeters (mm).^9^ Aneurysmal SAH is more common in women and in patients 40–60 years old.

Aneurysms typically present at cerebral artery bifurcation points in both anterior or posterior regions. Aneurysmal pathophysiology has been theorized to involve congenital weakness in the vessel wall, or degenerative changes resulting in destruction of elasticity of the vessel wall at points of high turbulence such as bifurcations.^10^

### Classification

There are several systems of classification for SAH. The Hunt and Hess score and World Federation of Neurological Surgeons grading system are both used to predict patient outcome, and the Fisher grade helps to predict vasospasm. Given the retrospective derivation of these scales and little if any assessment of intra- and interobserver variability, no single scale can be recommended over others.^11^ In terms of patient-centered outcomes and prognosis, specific scores were not seen to perform any better than the Glasgow Coma Scale (GCS).^12^

The classification systems do, however, help highlight an important concept of *spectrum bias*. As we delve into the diagnosis of SAH, it is important to note that some patients with SAH, for example Hunt and Hess grades I and II patients, are more commonly missed because symptoms are milder, and they may have smaller aneurysms with less subarachnoid blood. These patients do *not* necessarily do better or have less morbidity with rupture or re-rupture.

### Diagnosis

The diagnosis of SAH should be considered in any patient with a severe and sudden onset or rapidly escalating headache. With such a large number of patients presenting to the ED with a chief complaint of headache, differentiating those with a benign cause from those with an emergent etiology such as SAH can be difficult. Deciding which patients require a workup for SAH is often the most challenging part of the emergency physician’s care, in part due to the low frequency and high acuity of the illness.

Classic teaching characterizes the headache of SAH as a “thunderclap headache,” which is defined as a sudden, severe headache often described as the worst of the patient’s life.^14^ The headache is typically a sudden onset, which is commonly characterized as occurring within a few minutes, although research parameters include headache that reaches maximum intensity within one hour. Symptoms that increase the likelihood of a subarachnoid bleed as the cause of headache include exertional onset, syncope, vomiting, neck pain, and seizures.^15^ Focal neurologic deficits, meningismus, and/or retinal hemorrhage may be present, but up to 50% of SAH patients have a normal neurologic exam.^16^ Recent research has attempted to shed light on which elements of the history and physical exam are correlated with and discriminating for the diagnosis of SAH.

**Figure 1:**
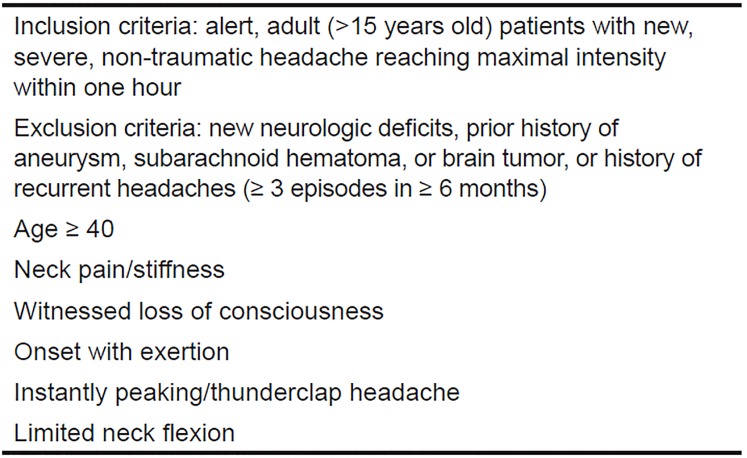
Ottawa subarachnoid hemorrhage decision rule.

Perry et. al published the Ottawa SAH Rule in 2013 after prospectively assessing 2131 adult patients with a non-traumatic headache that reached maximum intensity within one hour (Figure 1).^17^ Subjects were excluded if they had a pattern of similar headaches, had papilledema, or focal neurologic deficits on exam, or had a prior history of aneurysm, SAH, neoplasm, or hydrocephalus. Of the 2,131 patients investigated, 132 were ultimately diagnosed with SAH, giving a prevalence of 6.2%. The authors describe a decision rule with 100% sensitivity, although the specificity is at best 15% (Table). By this rule, any one criterion suggests that the patient should get a full workup. The low specificity, however, can have the deleterious effect of increasing the number of patients who undergo full workups, and are subsequently exposed to unnecessary radiation, procedures, and perhaps invasive procedures. While the merits of the Ottawa decision rule can be argued, it has helped delineate which historical elements, signs, and symptoms are statistically correlated with a confirmed diagnosis of SAH. Given that one of the most difficult elements of a SAH diagnosis is determining in whom a workup is needed, these data can inform the clinician’s process of determining pretest probability, even if the rule is not used in its entirety.

### Diagnostic Tools

#### Computed Topography

When a clinical suspicion for SAH exists based on history and physical exam, non-contrast computed tomography (CT) is the first diagnostic tool. It is also valuable in excluding other pathologies such as intracranial hemorrhage, malignancy, or abscess.

#### Timing of Computed Tomography

At the onset of the bleed, subarachnoid blood is the most readily visible on CT, but it becomes more difficult to appreciate as red blood cell (RBC) degradation progresses. Advances in neuroimaging have increased the sensitivity of non-contrast CT, raising questions regarding the need for lumbar puncture (LP) in the face of a negative CT.

A meta-analysis published in 2016 attempted to answer the question of CT sensitivity with relation to time from symptom onset.^18^ The analysis, which included five studies, assessed patients with a thunderclap headache and normal neurologic exam. While the results carry many of the limitations of a meta-analysis, a conservative statistical analysis showed that a non-contrast CT completed within six hours of headache onset had a sensitivity of 98.7% with confidence intervals 97.1%–99.4%. The authors took into consideration the following criteria: patient must have a hematocrit > 30% and an isolated thunderclap headache without seizure, syncope, or neck pain; and the CT image must be third generation or newer, of high quality, read by an attending-level radiologist, and evaluated with the indication for imaging being thunderclap headache or concern for SAH. If these criteria are met, many consider a negative head CT within six hours to be a “rule-out” study given the sensitivity and confidence intervals.

#### Lumbar Puncture

If non-contrast head CT is not definitive (time to study, patient elements [i.e., severe anemia], interpretation limitations [i.e., trainee radiologist, motion artifact], etc) the next recommended diagnostic tool is the LP. In these instances the LP is looking for two elements that raise the concern for SAH: 1) RBCs; and 2) xanthochromia (bilirubin in cerebrospinal fluid [CSF]).

Given the sensitivity of the CT discussed above, shared decision-making should be conducted with regard to LP. In particular, with sensitivity of near 99% for an adequate study if completed within six hours, and meeting the criteria outlined above (Dubosh), patients should be made aware of the low diagnostic utility of LP if completed after a CT.^19^ In this setting, risks (adverse events and false positives) generally outweigh benefits and LP is advised against. There are rare instances in which the clinical scenario so strongly suggests SAH that even an adequate negative CT completed within six hours is unable to rule out SAH and should be followed by LP. If the imaging is completed after the six-hour timeframe, the sensitivity of CT drops to 85.7%. In these cases, the diagnostic utility of LP increases as the probability of SAH after negative CT also increases. In such cases, LP is indicated. It should also be noted that, in keeping with the low prevalence of this disease, one recent study showed a roughly 0.4% of LPs revealed aneurysms.^20^ Shared decision-making is still recommended, as with any invasive procedure.

#### Red Blood Cells

Intact RBCs will be seen early in the course of the disease and decrease as the cells break down and are resorbed. Fitting the pathophysiology, the presence of RBCs in the fourth tube of CSF is thought to represent SAH. Unfortunately, a LP is often a technically difficult procedure and rates of “traumatic tap,” or introduction of erythrocytes by local trauma and needle manipulation can approach 30%.^21^ This complicates the diagnosis of SAH by RBC results. Because differentiating between a true SAH and a traumatic tap is of the utmost clinical importance, authors have researched criteria to help differentiate the two.

Perry et al. published data comparing LP results in patients with SAH (by research gold-standard confirmation) to those without that final diagnosis, most notably patients with a traumatic tap without concurrent SAH.^21^ In this analysis, the researchers found that setting a cutoff of 2,000 × 10^6^ RBCs per liter (L) in the final CSF tube combined with no xanthochromia, irrespective of RBCs in the first tube, captured all patients with a final diagnosis of SAH while excluding most patients with a traumatic tap. Patients were considered to have a SAH if they had any of the following: CT head positive for blood in the subarachnoid space; xanthochromia on LP; or RBCs of 2,000 × 10^6^ in the final tube of CSF with an aneurysm on CT angiography (CTA) that required neuro-intervention or resulted in death. To our knowledge, this fourth-tube cutoff for diagnosis of SAH has not yet been incorporated into professional society guidelines. Generally, it is believed that a traumatic tap produces a lower RBC count and possibly a more rapidly diminishing count from tube one to four.^22^ Multiple authors have shown that the approach of comparing the first and fourth tubes is unreliable, in light of the fact that traumatic tap and SAH are independent entities.^21^,^23^

#### Xanthochromia

True xanthochromia is pathognomonic for SAH. This is valuable when there is high clinical suspicion and RBC count is not sufficiently elevated to differentiate from a traumatic tap diagnostic. Xanthochromia is detected either by visual inspection of the CSF tube vs a tube of water, or by spectrophotometry. RBCs that have shed into CSF from SAH will ultimately break down and release oxyhemoglobin, which then converts to bilirubin in vivo, interpreted as xanthochromia, or literally “yellow color.” It should be noted that blood from a traumatic tap can produce oxyhemoglobin when exposed to natural light, which can produce a yellow color, but since it is outside the body it will not produce bilirubin.^24^ Protecting the specimen from light will minimize the conversion of RBCs to oxyhemoglobin. Alternatively, spectrophotometry can differentiate the oxyhemoglobin of traumatic tap from the bilirubin of SAH. Visual inspection, however, is still used in most institutions.

#### Timing

As with CT, controversy and practice variations exist with respect to timing of the LP. However, given the timing of RBC breakdown, the presence of any xanthochromia is delayed and most conservative estimates state an “up to 12 hours” timeframe.^25^–^27^ In pursuit of xanthochromia, some have historically advocated a delayed LP approach, typically 12 hours from ictus, but the literature supporting this approach used spectrophotometry, which is not available in most labs in the United States.^28^ No literature supports waiting for 12 hours to perform LP.^29^ Given the flow of ED care and desire to expeditiously diagnose SAH, it is reasonable to obtain CT and then immediate LP (if needed), with attention to xanthochromia supplemented by the RBC cutoff criteria as needed. If the LP shows > 2000 × 10^6^ RBCs in tube four, standard practice is to follow this with a CTA to assess for aneurysm. Conversely, if the cell count is < 2000 × 10^6^/L and no xanthochromia is seen, then SAH is ruled out.

In the Perry study four out of five sites used visual inspection for xanthochromia, and 39% of all LPs were done within 12 hours of headache onset. Considering this, and the fact that results were confirmed with blood in subarachnoid space on CT, xanthochromia or RBCs in the final tube, and an aneurysm by cerebral angiography requiring neurovascular intervention or resulting in death, we believe that visual inspection is not only the most-often used modality to determine xanthochromia, but is reasonable for this purpose. Often, there is lack of clarity on exact time of ictus, and more importantly we have no pathophysiologic data showing a standard timeframe for the processes of RBC degradation into xanthochromia. Given the pathognomonic characteristics of xanthochromia, these authors (JH + EM) recommend that CSF samples be analyzed for both RBC count and xanthochromia regardless of timing of LP.

#### Computed Tomography Angiography

Over the last decade, CTA of the brain has become part of the discussion in ruling out SAH. As a non-invasive means of highlighting vascular anatomy and detecting aneurysms, CTA has many advantages. Much like non-contrast head CT, advances in neuroimaging have shown CTA to have a sensitivity of up to 98% and a specificity of 100% for aneurysms in patients with known SAH. These statistics are derived from a small data set (n= 65) where CTA results were compared to gold standards of digital subtraction angiography or surgical findings.^30^,^31^

Some propose CTA as an alternative to LP after a negative non-contrast CT.^19^,^31^ With the prevalence of aneurysms estimated to be ~2–5% in the general population,^8^ there is a concern for incidental findings and false positives. An aneurysm found on CTA may be incidental and unrelated to the cause of headache. For example, a patient with a moderate pretest probability of SAH on presentation at 12 hours of symptoms is generally not thought able to be ruled out by non-contrast head CT given its sensitivity of ~85%. Some advocate that if the patient has a CTA that is negative for aneurysm after a negative non-contrast head CT, this is accepted as being conclusively negative for SAH.^19^ In addition, if it is not possible to perform LP for any reason, such as coagulopathy, the CTA could be used, with acknowledgment and consideration for its limitations.

**Figure 2:**
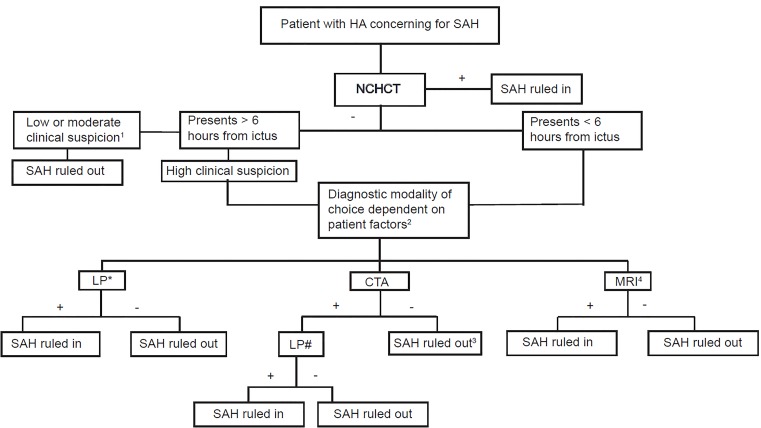
Algorithmic assessment for SAH in patient with sudden onset severe headache. *HA*, headache; *SAH*, subarachnoid hemorrhage; *NCHCT*, non-contrast head computed tomagraphy; *LP*, lumbar puncture; *CTA*, computed tomography angiography; *MRI*, magnetic resonance imaging. ^1^With criteria met for Perry study [Perry et al. BMJ 2011]^47^ ^2^Patient factors include anticoagulation status, patient willingness to undergo LP, history of lumbar spinal fusion or other surgery, and time from ictus (with longer time favoring MRI) ^3^Caveat for this strategy includes the potential to miss aneurysms < 4 millimeters ^4^MRI is an acceptable diagnostic at > 24 hours from ictus, prior to this sensitivity is lacking. *This is the recommended strategy by AHA/ASA, ACEP, and these authors ^#^Recommended to decrease the false positive rate of CTA.

Based on best available literature, a CTA without findings of aneurysm when coupled with a negative non-contrast head CT has a post-test probability of disease of < 1%.^31^ This percentage is important because it falls below most clinicians’ *test threshold*, which is the probability of disease below which no further investigation is required. However there is one confounding factor in this suggested algorithm (Figure 2). The sensitivity of CTA is 92.3% for aneurysms < 4mm,^32^ and in contrast to pathologies where the size of the lesion correlates with the severity of disease (i.e., pulmonary embolus), a small, ruptured cerebral aneurysm can still lead to significant morbidity and mortality.

Collectively, for patients in whom a CT is completed at > 6 hours, a CT-CTA approach is pursued by some, but has limitations, most notably, the finding of incidental aneurysms and inability to detect small culprit aneurysms. If the CTA is positive for aneurysm, completing an LP at that time to determine incidental vs symptomatic could be considered. Limitations to this approach include radiation dose to patient, contrast dye exposure, and detriments to department flow of such an algorithm. As noted above, this approach of CT-CTA carries a low sensitivity for small but symptomatic aneurysms.^19^ If this approach is used, the limitations and risk of false positive results should be discussed with the patient in a shared decision-making process.

#### Magnetic Resonance Imaging

Magnetic resonance imaging (MRI) can be used to assess for SAH, with certain limitations. The challenge with using MRI for SAH is that the blood is combined with CSF that has a high oxygen concentration, thus delaying the transition of blood products to a deoxyhemoglobin state that is better imaged with MRI.^33^ Since there are no data showing a discrete timeframe for the use of MRI, the decision to use MRI to assess for blood should be used in consultation with radiology and neurology or neurosurgery. The combination of fluid-attenuated inversion recovery and susceptibility-weighted imaging has been shown to be 100% sensitive for SAH, although most cases were imaged greater than 24 hours after the ictus of headache.^34^ If MRI is negative for SAH, LP is still recommended.^1^,^35^,^36^ Magnetic resonance angiogram is 95% sensitive for aneurysms > 3 mm.^37^ With all of these limitations, MR imaging is not recommended as a primary imaging modality, but may be useful in certain atypical cases, in particular in patients with a long delay from ictus to presentation.

### Summary of Available Diagnostic Tools

Many tools are available to assess for SAH including non-contrast CT, LP, CTA, and MRI. Understanding the potentially high mortality in the case of a missed SAH should mandate a diagnostic strategy with the highest sensitivity possible, which is currently accepted to be non-contrast CT followed, if negative, by LP.^1^,^31^,^34^ This is the algorithm supported by both the American Heart Association (AHA) and American College of Emergency Physicians (ACEP). This strategy, of course, should take into account the previously described limitations of the LP. While CT/LP remains the most accepted rule-out method, other approaches do exist. Many practitioners have accepted the recent literature showing non-contrast CT to be an acceptable stand-alone study if completed within six hours.^18^ If using any of the other tools described above, we must appreciate and work within the known limitations of each method.

### ED Management

Once the diagnosis of SAH is established, the most important time-sensitive goals include confirmation of airway security and stabilization of hemodynamics. Intubation should be undertaken in the setting of low Glasgow Coma Scale Score or inability to protect the airway, but care should be taken to mitigate increases in mean arterial pressure (MAP) during the intubation process. This can be accomplished through careful choice of sedation agents for rapid sequence intubation and push-dose vasoactive agents if blood pressure does become elevated. Cardiac monitoring is important, as patients with devastating brain injury are at risk for neurocardiogenic stunning.^38^

The next priorities are to reduce systolic blood pressure (BP) and reverse anticoagulation to mitigate the risk of aneurysm re-rupture. Specific BP goals are unclear and need to be weighed against the risk of ischemia or infarction with hypotension. Guidelines recommend targeting BP < 160 systolic,^35^ although many consider lower targets of 140–150. Nicardipine (5 milligrams per hour (mg/h) intravenous (IV), may increase by 2.5 mg/h q5–15 minutes (min); Max: 15 mg/h), labetalol (40–80 mg IV q10 min, start 20 mg IV × 1; Max 300 mg/total dose; Alt: 2 mg/min IV), and clevidipine (4–6 mg/h IV, start 1–2 mg/h IV, double rate q 90 seconds until near BP goal, then increase. By smaller increments q5–10 min; max:32 mg/h) are effective agents, often used in infusion form to avoid hypotension. In the setting of bradycardia, hydralazine may also be used. Nitroprusside and nitroglycerin should be avoided due to their significant vasodilatory effect and the risk of increasing intracranial pressure (ICP).

Reversing anticoagulation should be accomplished as soon as possible. Vitamin K antagonists can be reversed with phytonadione (vitamin K) and 4-factor prothrombin complex concentrate (PCC) or fresh frozen plasma. PCC is preferable as it has a more rapid onset, does not need to be thawed or blood-type matched, and can be infused rapidly with less volume and risk of fluid overload.^39^ Antiplatelet agents should be reversed with platelet infusion, and desmopressin should be considered.^40^ The utility of platelet administration has been questioned recently after a recent trial showed increased mortality with platelet infusion for patients taking antiplatelet therapy.^41^ This trial, however, studied patients with spontaneous intracerebral hemorrhage, a different pathophysiology than SAH, and generalization of the results is not directly applicable.

Direct thrombin inhibitors such as dabigatran can be reversed with idarucizimab, which is United States Food and Drug Administration (FDA) approved and widely available. Andexanet alpha, an antidote for Factor Xa inhibitors (apixaban, edoxaban, rivaroxaban) is FDA approved for reversal of major bleeding with apixaban and rivaroxaban and available on a limited basis (Young).^39^ If the patient with SAH is taking any Factor Xa inhibitor, including unfractionated heparin or fondaparinux, PCC is recommended as a first-line agent for reversal, unless Andexanet alpha is indicated and available.

Regardless of anticoagulation mechanism, a pre-approved institutional protocol should be in place for rapid utilization, with input from hematology, blood bank, emergency medicine, and neurosurgery in order to most efficiently reverse anticoagulation. Other strategies to reduce risk of aneurysmal re-rupture are targeted toward controlling pain, nausea, and valsalva effect by treatment with analgesics, antiemetics, and stool softeners as needed. Fentanyl is a very effective and easily titratable analgesic, and is quickly titrated off to facilitate neurologic exams. Nimodipine, a calcium channel blocker used to improve outcome in SAH patients can be started in the ED, with caution given to the patient’s ability to swallow and the potential to inappropriately reduce BP.^35^ Other best practices include arterial-line BP monitoring, crystalloid to target euvolemia, and head of bed at 30° to protect against aspiration and to allow jugular venous outflow for ICP protection.

Many patients with SAH will require ventriculostomy drainage, either for hydrocephalus or periprocedurally to help with ICP complications. Antiepileptic medications may be recommended if the neurologic exam is poor, or the amount of blood is significant, portending risk of clinical or subclinical seizure. There has not been a definitive study to recommend any specific antiepileptic agent, as each has therapeutic benefits and risks and is ideally tailored by the patient’s profile. The most common agents are phenytoin (load 10–20mg/kilogram [kg] IV max: 50mg/min), fosphenytoin (10–20 phenytoin sodium equivalent (PE)/kilogram (kg) IV; infuse slowly over 30 min; max: 150mg PE/min) and levetiracetam (15–20mg/kg over 30 min).

These therapeutic modalities should be discussed with the admitting neurointensivist or neurosurgery team. Continuous electroencephalogram (EEG) monitoring may be started in the intensive care unit (ICU).

The ultimate therapeutic goal, once a bleeding aneurysm is identified, is to secure it surgically by coiling or clipping. While coiling is the preferred method,^42^ since it is less invasive than open surgical clipping, data are inconclusive as to whether long-term outcomes are better with either procedure, but guidelines suggest that coiling should be performed if both are possible.^43^,^44^ In some cases, tortuous vascular anatomy or other contraindications to coiling make open surgery necessary. Earlier treatment and securing the aneurysm is associated with lower risk of rebleeding.^44^ In the event that surgical treatment is delayed, antifibrinolytics such as aminocaproic acid may be used for a short period of time to mitigate the risk of re-rupture. Tranexamic acid and prothrombin complex concentrates have not been studied in this setting. This treatment modality is not backed by evidence, invokes risk of thrombosis, and is best discussed with the neurosurgery team.^44^,^45^

Once the aneurysm is secured, the greatest risk to patient outcome is that of vasospasm and delayed cerebral infarction (DCI). Many strategies are employed to assess for vasospasm, including hourly neurologic exam, strict euvolemia, continuous EEG, transcranial Doppler, permissive or induced hypertension, electrolyte monitoring and CT or direct angiography. If vasospasm is detected, timely treatment is paramount to decrease the risk of associated DCI. Treatment can be catheter-directed calcium channel blocker administration, such as nicardipine or verapamil, or vessel angioplasty.^46^

Our patient had a non-contrast CT 10 hours after onset of headache, which was negative for blood but positive for mild hydrocephalus. Hydrocephalus presents in 20–30% of SAH patients, and is generally thought to be a result of fibrotic changes associated with inflammatory reaction to blood at the arachnoid granulation. This can be suggestive of a pathologic process but is not diagnostic of SAH. The patient then consented for a LP, which showed RBCs in tubes 1 (14,000×10^6) through tube 4 (13,500×10^6). She was started on a nicardipine infusion for BP management and was given fentanyl for pain control. Neurosurgery was consulted, and she was admitted to the neuro ICU for hourly neurologic examinations and preparation for coiling the next day.

Many centers have access to neurosurgical coiling capability, as part of a comprehensive stroke center designation, but in some areas surgical clipping may be the only available procedure. Coiling is typically preferred, having shown better outcomes in the long run, but in some cases patient anatomy (tortuous vessels or plaque in carotid arteries) may preclude this procedure and the patient may need to be transferred to another center.^42^ Ventriculostomy placement prior to transfer will depend on the presence and severity of hydrocephalus, and should be discussed with the neurosurgery team.

The assessment and treatment of SAH is a dynamic and changing field, with the advent of advanced imaging, better understanding of pathophysiology and improved surgical techniques. SAH is rare but can be a devastating occurrence. Understanding the pathophysiology, demographics and risk factors helps to accurately evaluate the patient who presents to the ED with sudden-onset severe headache. The diagnostic strategy is key to decide which patients warrant full workup.

CT followed by LP is the standard diagnostic strategy, as per guidelines from ACEP, AHA and ASA, but many other advanced imaging options have come to the fore, making it important to understand the benefits and limitations of each diagnostic tool. Diagnostic sensitivity is critical, as a missed diagnosis of SAH can lead to increased mortality if the aneurysm re-bleeds. Shared decision-making can ensure that each patient understands risks and benefits. Disease recognition and prompt diagnosis is the primary responsibility of the emergency physician, while patient-specific management decisions are best made in a multi-disciplinary fashion.

## CONCLUSION

Despite advances in the diagnosis and treatment of aneurysmal subarachnoid hemorrhage, mortality remains high. We are indebted to scholars who have contributed to the growing body of knowledge around aneurysmal SAH and appreciate that there is much more work to be done for this devastating disease.

## Figures and Tables

**Table:** Hunt and Hess grading for subarachnoid hemorrhage.^13^

Grade	Criteria	Survival
I	Asymptomatic or mild headache with slight nuchal rigidity	70%
II	Moderate to severe headache, nuchal rigidity, no neurological deficit other than cranial nerve palsy	60%
III	Drowsiness, confusion, or mild focal deficit	50%
IV	Stupor, moderate to severe hemiparesis, possibly early decerebrate rigidity or vegetative disturbance	20%
V	Deep coma, decerebrate rigidity, moribund appearance	10%
